# Multifractal investigation of Ag/DLC nanocomposite thin films

**DOI:** 10.1038/s41598-020-79455-z

**Published:** 2020-12-17

**Authors:** Ştefan Ţălu, Bandar Astinchap, Senour Abdolghaderi, Azizollah Shafiekhani, Ilya A. Morozov

**Affiliations:** 1 The Directorate of Research, Development and Innovation Management (DMCDI), Technical University of Cluj-Napoca, 15 Constantin Daicoviciu St., 400020 Cluj-Napoca, Cluj County Romania; 2 Department of Physics, University of Kurdistan, 66177-15175 Sanandaj, Iran; 3 Research Center for Nanotechnology, University of Kurdistan, 66177-15175 Sanandaj, Iran; 4 Department of Physics, Alzahra University, Vanak, 1953833511 Tehran, Iran; 5 Department of Education, Kurdistan Province, Sanandaj, Iran; 6 Institute of Continuous Media Mechanics UB RAS, 1 Korolev St., 614013 Perm, Russia

**Keywords:** Nanoscale materials, Structural materials

## Abstract

The objective of this study is the experimental investigation of the silver in diamond-like carbon (Ag/DLC) nanocomposite prepared by the co-deposition of radio frequency plasma-enhanced chemical vapor deposition (RF-PECVD) and RF-sputtering. Atomic force microscopy (AFM), X-ray diffraction analyses, ultraviolet–visible (UV–visible) spectroscopy measurements were applied to describe the three-dimensional surface texture data in connection with the statistical, and multifractal analyses. Additional information about structure–property relationships in prepared Ag/DLC nanocomposite was studied in detail to allow a better understanding of the surface micromorphology. The performed analysis revealed the studied samples have multifractal properties and can be included in novel algorithms for graphical representation of complex geometrical shapes and implemented in computer simulation algorithms.

## Introduction

In the last decades, considerable progress has been made in the development of theoretical and computational methods to characterize the silver in diamond-like carbon (Ag/DLC) nanocomposite microstructures at the nanoscale in both the academic world and industry, because these films offer a wide range of exceptional physical, mechanical, and tribological properties.

Manninen et al.^1^ studied the Ag–DLC coatings deposited by magnetron sputtering to evaluate: the Ag nanoparticle size distribution along the coatings thickness and the silver stability in DLC coatings.

Goto^2^ reported results concerning the characteristics and tribological properties of both Ag/DLC nanocomposite coatings and Cu/DLC nanocomposite coatings with hydrogen-free DLC matrix deposited by RF magnetron sputtering using a concentric composite target.

Wu et al.^3^ studied the doping effects of Ag concentration on microstructure, mechanical and vacuum tribological properties of the DLC films.

Ji et al.^4^ investigated trace Ag–Ti co-doped DLC films prepared on stainless steel and silicon (100) substrates by medium frequency unbalanced magnetron sputtering and described the effects of sputtering current of Ti target on the microstructure, mechanical and tribological properties.

Venkatesh et al.^5^ prepared Ag-doped hydrogenated carbon thin films by a hybrid PVD–PECVD deposition process and observed that the working pressure and CH_4_/Ar ratio played a key role in controlling the Ag content in DLC.

Abdolghaderi et al.^6^ deposited Ag-DLC on glass and silicon substrates by the co-deposition of RF-sputtering and RF-sputtering method and studied electrical percolation threshold in nanocomposites.

Stach et al.^7^ applied a stereometric analysis of 3D surfaces of the Ag–DLC nanocomposite films fabricated by Radio FrequencyPlasma-Enhanced Chemical Vapour Deposition (RF-PECVD).

Ţălu et al.^8^ investigated the effects of deposition time on the nanoscale patterns of Ag/DLC nanocomposite synthesized by RF-PECVD and found the fractal dimension decreases with the increase of the deposition times on samples.

Ţălu et al.^9^ studied the topographic uniformity, the fractal parameters of succolarity, the lacunarity coefficient, and the Shannon entropy of Ag/DLC composite synthesized by RF-PECVD for a better understanding of the structure–property correlation.

Baba et al.^10^ prepared Ag-containing DLC films by a process combining reactive magnetron sputtering with plasma source ion implantation and observed that the structure and tribological properties were affected by the Ag content of the DLC films.

Jastrzębski et al.^11^ reviewed previously published experimental data concerning mechanical and physicochemical properties of DLC coatings doped with Ag, Si, Cu, Ti, Ca, F and P as candidates for biomedical applications.

Cloutier et al.^12^ studied Ag–DLC prepared in a hybrid plasma reactor and proposed a mechanism for Ag–DLC growth by plasma.

Wen et al.^13^ reviewed many synthesis methods of DLC films, research theories and analysis methods applied in biomedical field.

A correct knowledge of the surface microtexture of thin films is a key factor to predict the suitability of the material for the proposed application^14–17^. The surface morphology describing the irregular surface geometry in terms of its space-filling ability can be estimated by fractal/multifractal analyses with a minimal set of fractal/multifractal parameters^18–22^.

Information on the micro/nano scale surface microtexture has important implications for optimal design and offers new possibilities for investigating precise topographic and spatial characteristics^23–26^. Atomic force microscopy (AFM) is a useful tool for sampling real surface heights to determine complex spatial characteristics of a given surface^27–30^. AFM combined with image processing techniques can be used to further extract particular patterns in three dimensional (3D) complexities^31–33^.

The present investigation attempts to explore insights into the multifractal nature of the Ag/DLC nanocomposite prepared by the co-deposition method of RF-PECVD and RF-sputtering using AFM analysis in connection with their surface morphology.

## Methods

### Materials and synthesis

The Ag–DLC nanocomposite films were fabricated by the co-deposition of RF-PECVD and RF-sputtering in a chamber.

The chamber consists of two electrodes with different area, a silver disc (4 cm diameter) was the smaller electrode as a powered electrode and the other electrode disc (a 13 cm diameter) holding the substrates was grounded via the body of the stainless-steel chamber. The distance between electrodes was 5 cm. A capacitance coupled RF system with a 13.56 MHz and 160 W power supply and bias voltage 80 V. Acetylene gas was used as reactive and bombarding gas in the RF plasma system. Deposition was done on glass and silicon substrates on the grounded electrode at room temperature. The chamber was evacuated to a base pressure of about 10^−3^ Pa before the deposition and then the pressure rises to 4 Pa pressure using only acetylene gas.

**Figure 1:**
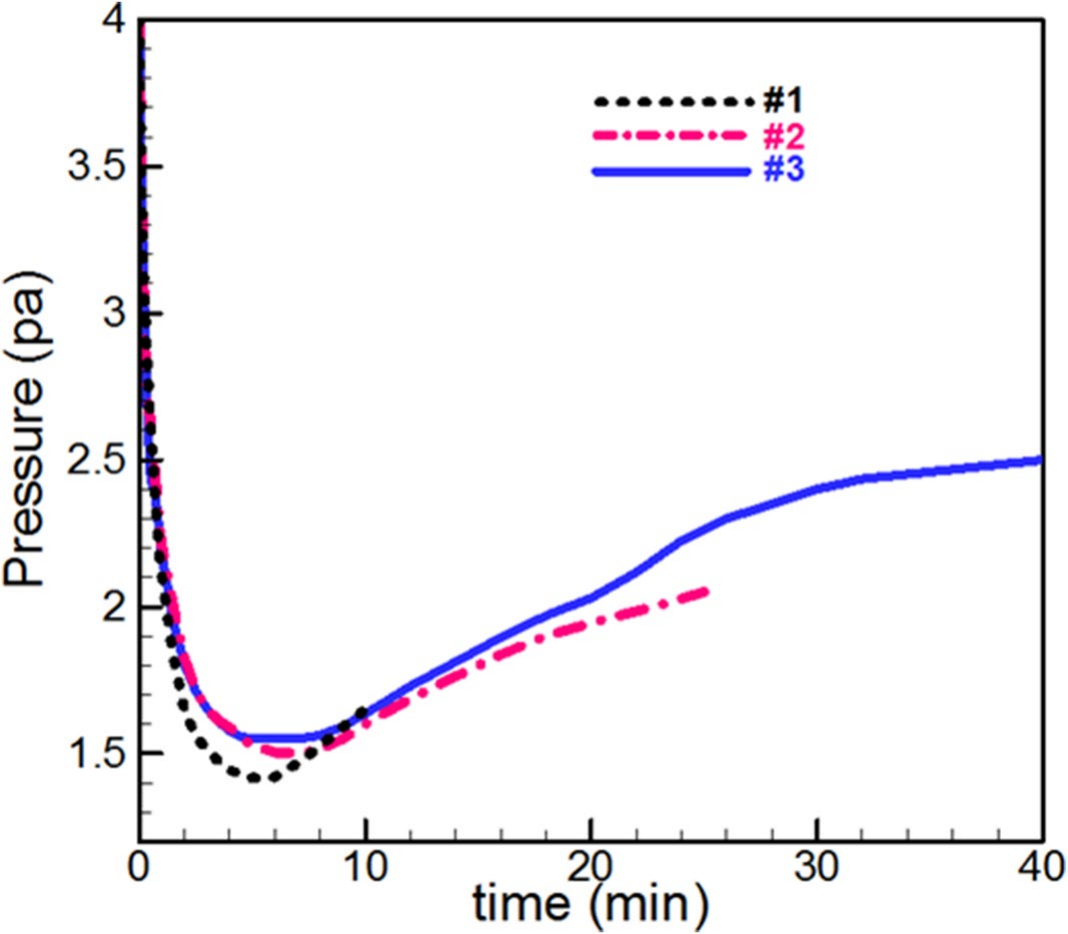
Deposition pressure diagram of the chamber (Pa) versus time of deposition (min) for samples #1 to #3.

The three samples #1, #2, and #3 were deposited on 1 cm × 1 cm glass and silicon substrates at different deposition times of 10, 25, and 40 min respectively. Chamber pressure changes in the sputtering process for samples are shown in Fig. 1.

As can be seen in the deposition diagram (Fig. 1) all three samples follow the same pressure reduction pattern. So that, at first pressure is decreased, which is due to the deposition of hydrocarbons from acetylene plasma, after that the pressure is increased and it's because of the presence of the silver atom in the chamber and its deposition in films.

Prepared samples were characterized by X-ray diffraction (XRD) (Philips analytical with CoKα X-ray source (λ = 0.154 nm)), UV–VISIBLE (Stellar-Net EPP2000 UVN-SR), and AFM with instrument model DMC DS 95 series in non-contact mode.

The multifractal analysis was applied to AFM images for the characterization of surface morphology.

## Results and discussion

### UV–visible spectroscopy measurements

**Figure 2:**
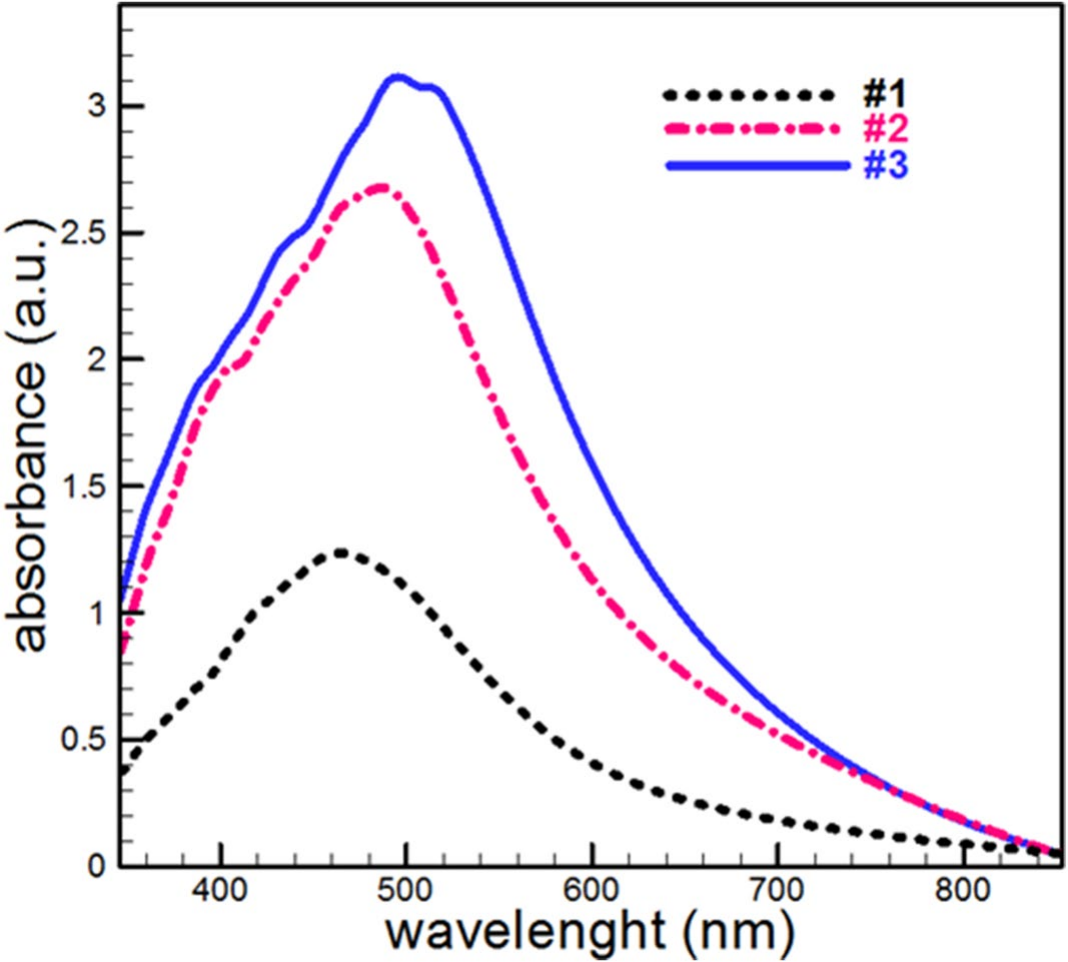
Absorption spectrum of fabricated samples.

UV–visible spectroscopy of samples in 350–850 nm range is shown in Fig. 2. It is observed that in the absorption spectrum for all three samples the peak appears. This peak is related to localized surface plasmon resonance (LSPR) of Ag nanoparticles, which confirms the presence of Ag nanoparticles in prepared samples.

The absorption spectrum shows the position of the LSPR peaks has changed with increasing deposition time. This redshift of plasmon peak represents an increase in particle size with deposition time. Scaling up and aggregation of silver nanoparticles (NPs) on the surface of thin films (sample #1 to #3) cause to symmetry broking of spherical silver NPs then quadrupole and multipole show up in the absorbance spectrum.

The Sp^2^/Sp^3^ ratio obtained from Fourier transform infrared (FTIR) spectroscopy is listed in Table 1. The peak in 2800–3100 cm^-1^ range is attributed to C–H, and C–C bonding, and the area of normalized Gaussian's peak related to the C–H, and C–C bonding amount.

**Table 1:** Obtained parameters from characterization of samples.

Sample	Deposition time (min)	Plasmon position Wavelength (nm)	Sp^2^/sp^3^	Resistance (× 10^−12^ Ω cm)	Size of NPs (nm)
#1	10	458.46	0.69	5.94	7
#2	25	517.30	0.15	5.52	15
#3	40	519.74	0.089	1.97	22

The obtained result shows that the sp^2^/sp^3^ ratio decrease with an increase in deposition time, which means the sp^3^ bond decreases, and the pseudo-diamond behavior of the samples is reduced. The sp^2^ bond is directly proportional to the number of silver nanoparticles in the nanocomposites. Based on this, sample #3 has silver NPs more rather than #2 and #2 more than #1^14^.

These results emphasize the previous discussion in UV–Vis analysis and deposition pressure diagram that the size (or amount) of the silver NPs increases with increasing deposition time.

### XRD analysis

To determine the crystalline structure of the specimens STOE-XRD diffractometer using Cu-Kα line (λ = 0.154 nm) was used.

**Figure 3:**
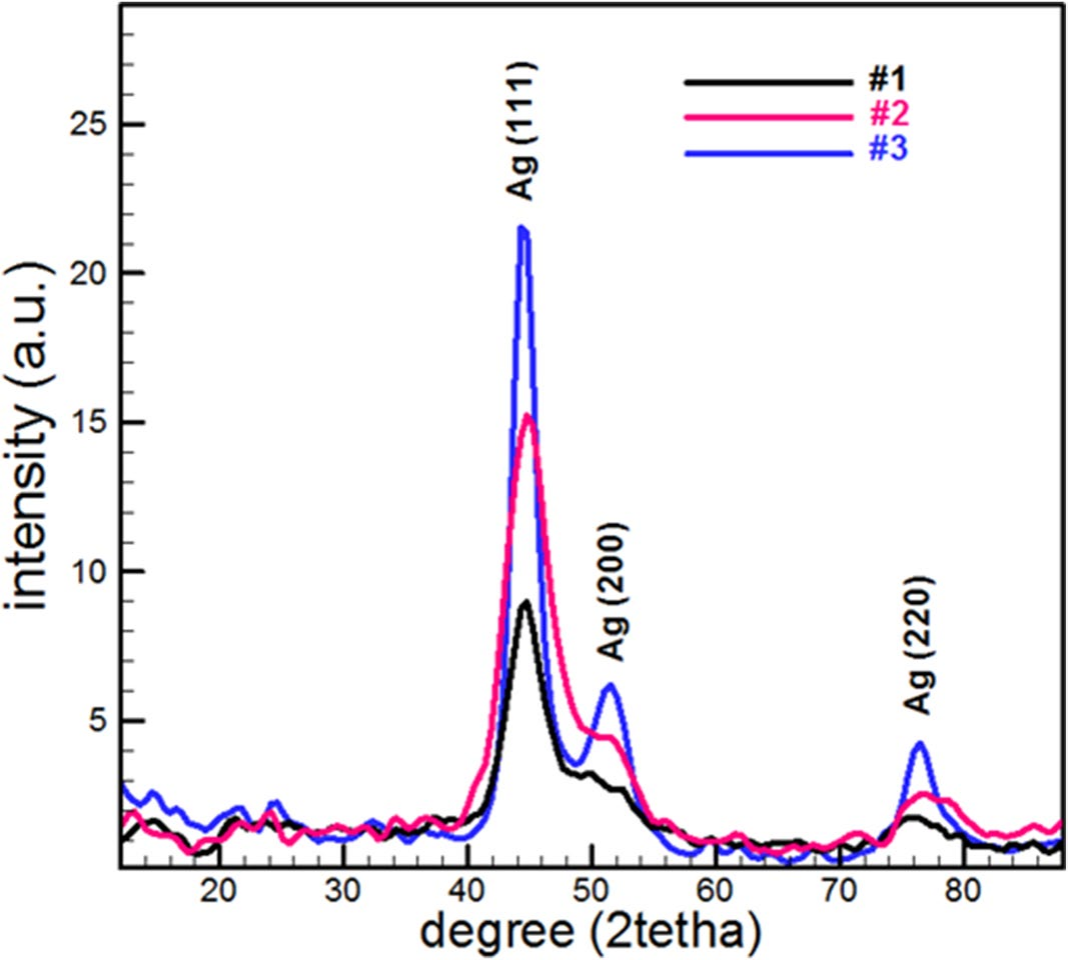
Typical XRD patterns of prepared Ag–DLC nanocomposites.

The XRD patterns of prepared samples are shown in Fig. 3. The crystals planes with related Miller indices correspond to peaks are determined in XRD patterns. Based on reference cards, all peaks that appeared in the XRD patterns are consistent with the peaks of silver nanoparticles with a Face-centered cubic structure. The increase of particle size with deposition time from Sherrer relation is obvious (Table 1).

Obtained results from XRD analysis are in good agreement with other analyses to confirm the presence of silver nanoparticles in thin films and increasing the size of that with deposition time.

### AFM measurements

The samples were measured by Veeco di CP-II Atomic Force Microscope in contact mode. CONT20A-CP tips were used for scan areas set on 0.5 μm × 0.5 μm size, with 1 Hz scan rate and 256 × 256 pixel resolution.

**Figure 4:**
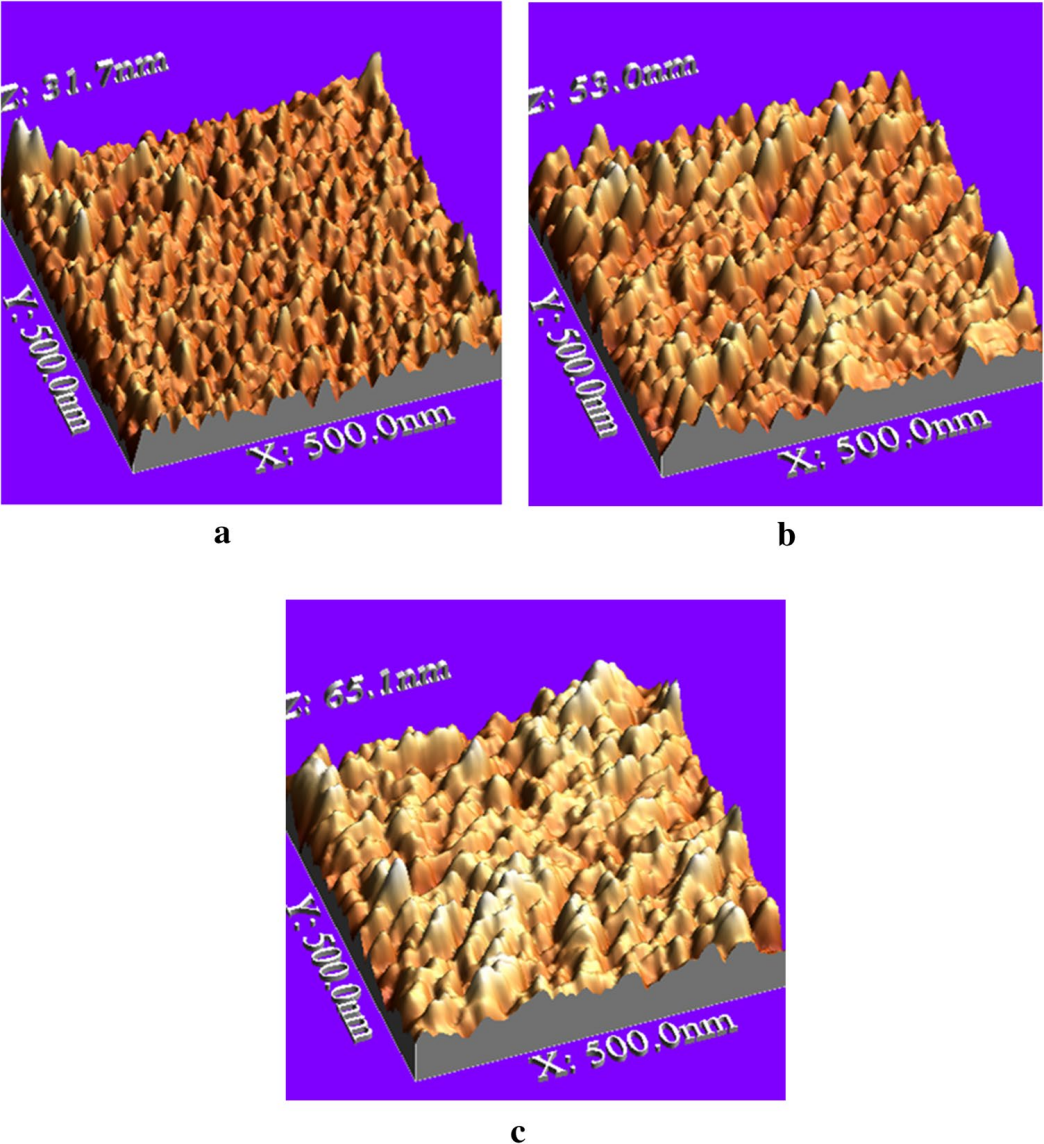
Representative AFM images of samples: (**a**) #1, (**b**) #2 and (**c**) #3 (WSxM ver 5.0 software, http://www.wsxm.es/).

**Figure 5:**
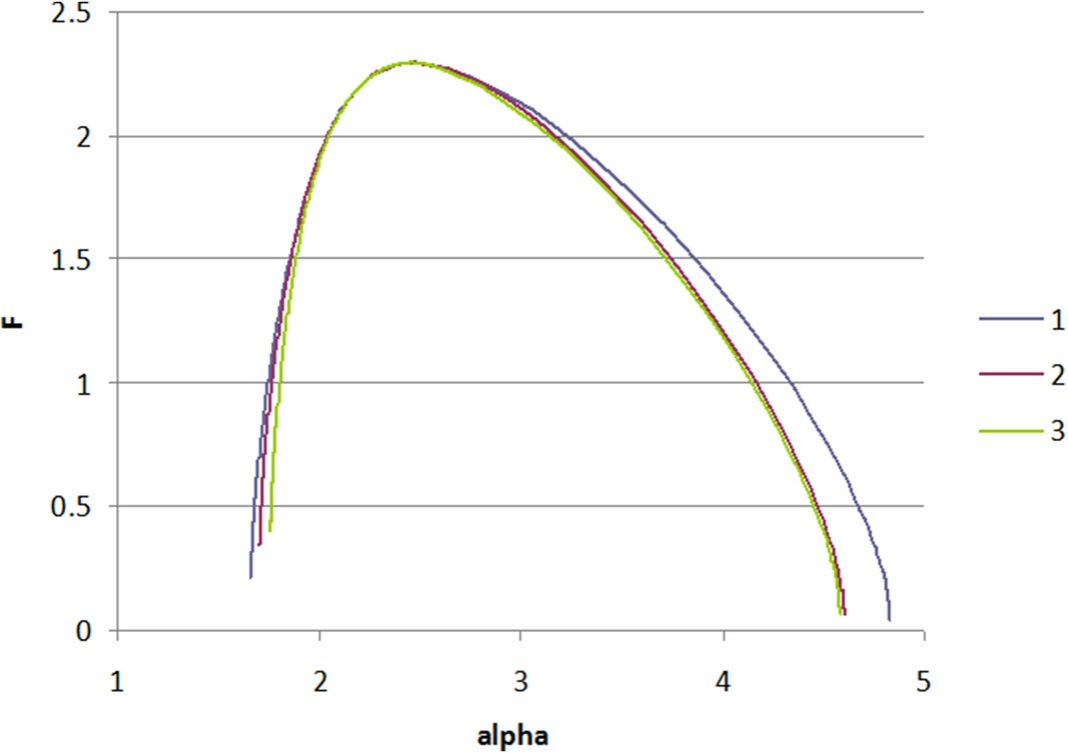
The graphical representation of RMS roughness of samples #1, #2 and #3 deposited at various times 10, 25 and 40 min.

For data analysis, the WSxM ver 5.0 software was used. Representative AFM images of rough substrates and their surface topography are shown in Fig. 4. The AFM pictures show whenever the deposition time is less the Ag nanoparticles are small in size and distributed almost uniformly and we have an almost regular network of particles embedded in DLC (Fig. 4a). Increasing the time of deposition, Ag nanoparticles distributed more and each site of particles became closer. In this stage of deposition close particles are associated or aggregated and form bigger particles with a large distance between newly formed ones. As one can see in Fig. 4b there is a small and big size of particles next to each other. In the next stage, the new small particles deposit and occupy a place in the distance between bigger particles (Fig. 4c). Particle size and location distribution changed relative with the time duration of deposition. Dependence of root mean square (RMS) roughness v.s. size of the observation window was calculated (Fig. 5).

### Multifractal analysis of AFM images

To characterize a multifractal system, we assume that the surface contains *N*(ε) quadrate cells, with side ε and *r*_*i*_(ε) = Σ*z*_*kl*_ is the cumulative fluctuation of height around the mean value in the *i*-th square. A statistical sum can be defined as^21–23^:

1 $$ Z(q,\varepsilon ) = \sum\limits_{i = 1}^{N(\varepsilon )} {p_{i}^{q} \left( \varepsilon \right)} \sim \varepsilon^{\tau (q)} , $$

where $p_{i}^{{}} \left( \varepsilon \right) = r_{i} \left( \varepsilon \right)/\sum\nolimits_{k = 1}^{N(\varepsilon )} {r_{k} \left( \varepsilon \right)}$ and the power exponent *q* ∈ (–∞; + ∞).

The generalized fractal dimensions can be expressed in function of the mass exponent *τ(q)* as:

2 $$ D_{q} = \tau (q)/\left( {q{-}{1}} \right). $$

The mass exponent *τ(q)* can be defined as:

3 $$ \tau \left( q \right) = q\alpha (q){-}f\left( {\alpha \left( q \right)} \right) $$

Equation (2) is usually written in terms of *D*_*q*_ as:

4 $$ \alpha \left( q \right) = d\tau \left( q \right)/dq. $$

**Figure 6:**
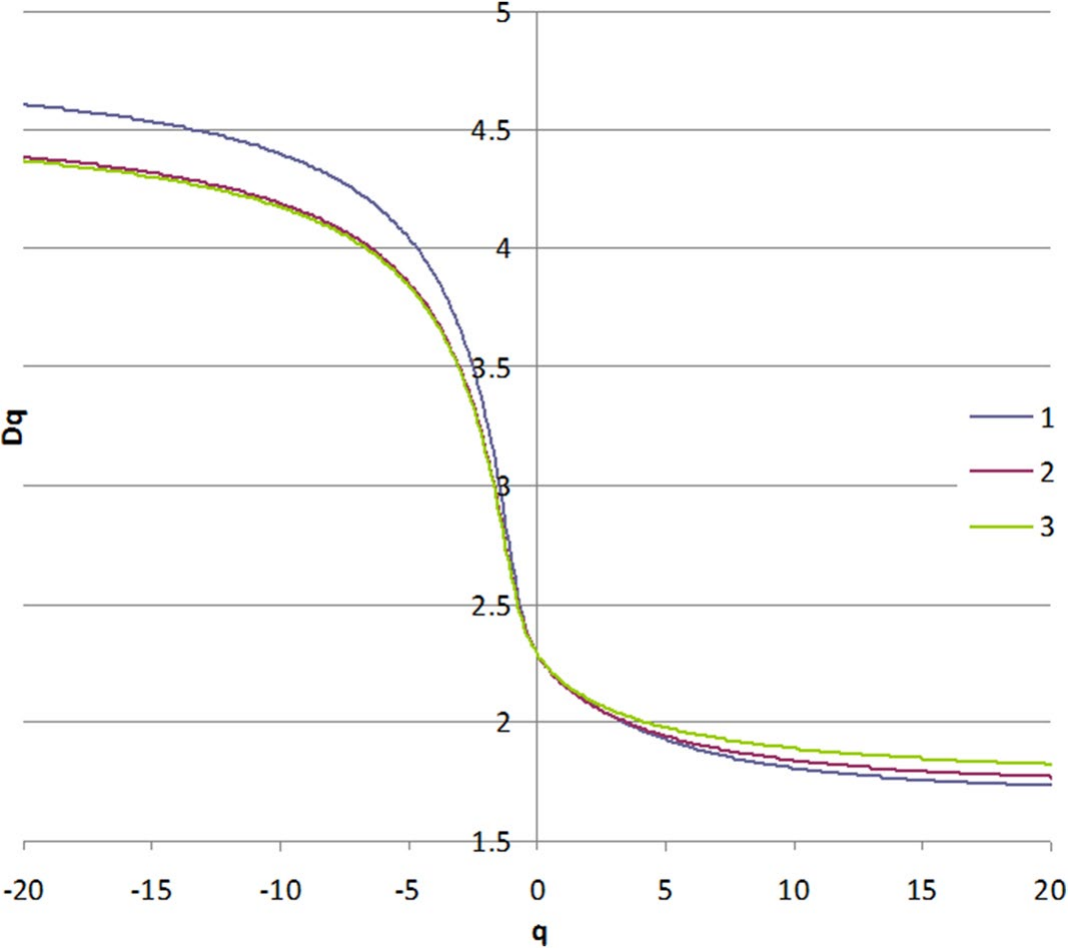
The graphical representation of the multifractal singularity spectra f(α) of samples #1, #2 and #3 deposited at various times 10, 25 and 40 min.

Multifractal singularity spectra *f*(*α*) shows the contribution of each monofractal in the whole structure for samples #1, #2, and #3 and represented by characteristic curves with a specific concave shape (non-linear and asymmetric form), and with different width spectrum *Δα*, (and invariably with two arms); these are graphically represented in Fig. 6. The left shoulder of the multifractal singularity spectrum *f*(*α*) is much shorter than the right one. It can be noted that the variability of shapes of the morphological objects can be estimated by the maximal height of the *f*(*α*) spectrum (given by the *D*_0_ value) and the multifractal width spectrum Δα = *α*_*max*_ *−* *α*_*min*_. *α*_*q*_ represents Hölder exponents of the *q*th order moment. In addition, for all samples, *Δα* > 0. Analyzing *f*(*α*) curves, we can note that sample #2 and sample 3# are very close, i.e. multifractal spectrum not changed. Sample #1 has a higher contribution of small-sized monofractals, i.e. small relief features with the given fractal properties.

**Figure 7:**
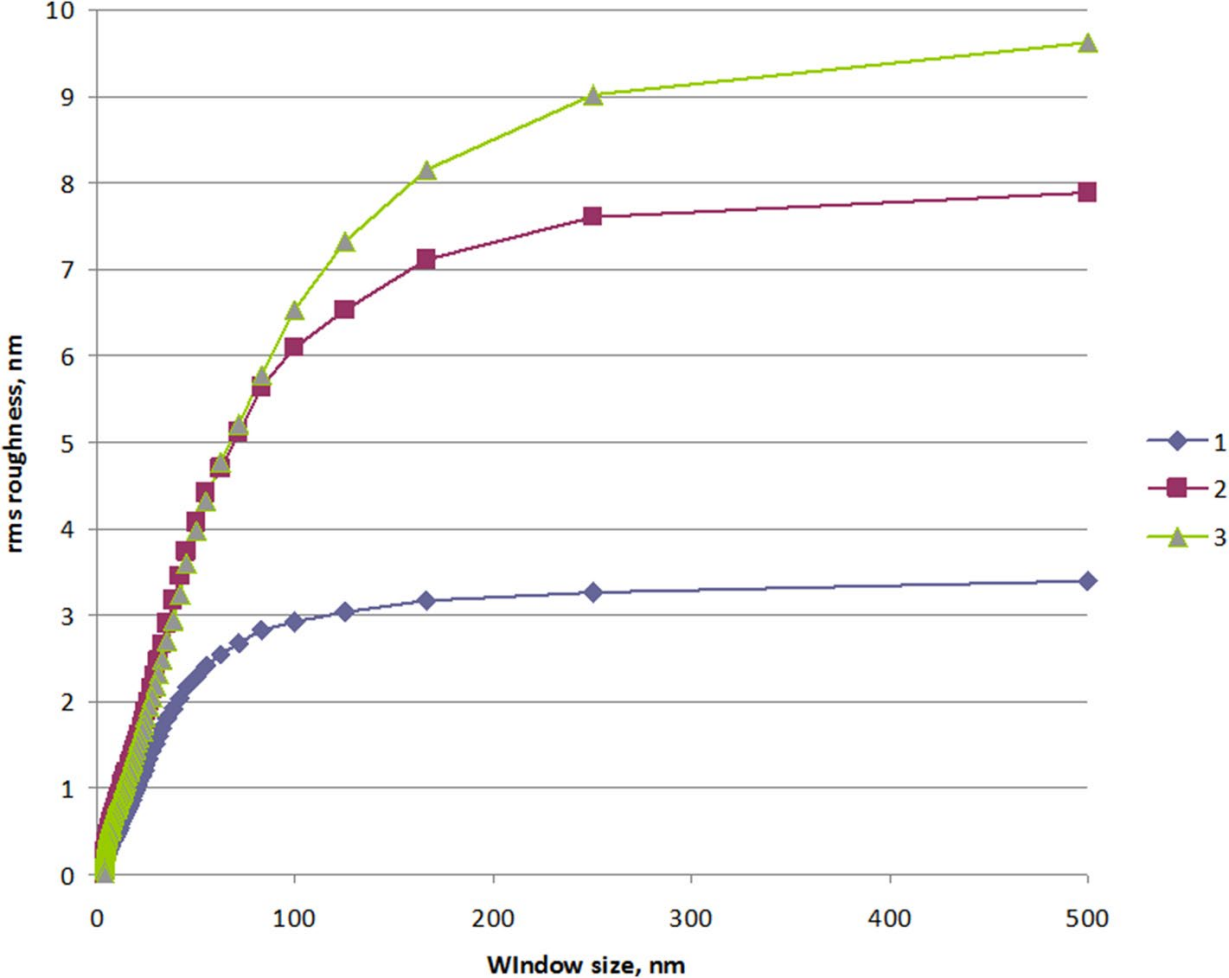
The graphical representation of the generalized fractal dimension Dq of samples #1, #2 and #3 deposited at various times 10, 25 and 40 min.

Generalized fractal dimensions *D*_*q*_ are shown in Fig. 7, with specific shapes of the *D*_*q*_ curves that are corresponding to typical multifractal surface morphology. The generalized fractal dimensions *D*_*q*_ was computed in the range − 20 ≤ *q* ≤ 20. On the other hand, the right shoulder of the multifractal singularity spectrum *f(α)* for the #1 sample is much longer than of all the other samples, respectively. Also, the left arm of the multifractal spectrum corresponds to strongly irregular areas, defined by a high dimension value, while the right arm of *f*(*α*) is associated with flat zones. These shapes revealed that the surface morphology substantially changed. It can be seen that for all the samples, the capacity (or box-counting) dimension *D*_*0*_ = 2.29.

The transition from a deposition time 1 to 2 (and 3) affected small peculiarities of relief: *D*_*q*_ for #1 lies higher at *q* < 0 than for #2 and #3.

For samples #2 and #3 the deposition time does not affect small-scale features: at q < 0 these graphs *D*_*q*_ are close; some deviations observed at *q* > 0, i.e. treatment slightly affected only large features of the surface.

Analyzing *f*(*α*) curves, we can note that #2 and 3# are very close, i.e. multifractal spectrum not changed. #1 has a higher contribution of small-sized monofractals.

The highest non-uniformity is observed for the sample with a deposition time of 10 min (#1) and lowest for the sample with a deposition time of 40 min (#3).

All this means that multifractals nucleate at a small deposition time, further treatment increases the size of objects without changing the multiscale properties.

### Statistical functions applied in the analysis of AFM images

A surface can be described by a height function, *h*(*r*) ≡ *h*(*x,y*) where the height is measured from a fixed reference frame, and *r* = (*x,y*) is any point on the surface.

The connection or correlation of spatially separated surface points can be computed by the autocorrelation (ACF) or height–height correlation (HHCF) function. The correlation length expresses the distance up to which the height of two surface points can be considered to be correlated.

The auto-correlation function, *R*(*r*), is defined as^14,34^:

5 $$ R(r) = \frac{1}{{w^{2} }}\frac{1}{{L^{2} }}\int_{ - L/2}^{L/2} {\int_{ - L/2}^{L/2} {\left[ {\left\{ {h(x,y) - \overline{h} } \right\}\left\{ {h(x + x^{\prime},y + y^{\prime}) - \overline{h} } \right\}} \right]} } dxdy $$

where *r* is the spatial separation between two arbitrary surface points, *r*_1_ = (*x,y*) and *r*_2_ = (*x* + *x́*, *y* + *ý*).

For a truly random rough surface, *R*(*r*) decays to zero with increasing *r*. The decay rate depends on the randomness of the surface.

The lateral correlation length, *ξ*, is defined as a length scale over which the magnitude of *R*(*r*) decreases to 1/*e* of its value at *r* = 0, i.e., *R*(*ξ*) = (1/*e*) *R*(0) spatial length over the surface at which this autocorrelation function drops down to 1/*e*. If the distance *r* between two surface points is within lateral correlation length *r* < *ξ*, the heights at these two points can be considered to be correlated. If the distance *r* between two points is greater than *ξ* the height at these two points is considered to be independent of each other.

The height–height correlation function, *G*(*r*), is defined as^14,34^:

6 $$ G(r) = \frac{1}{{L^{2} }}\int_{ - L/2}^{L/2} {\int_{ - L/2}^{L/2} {\left[ {h(x + x^{\prime},y + y^{\prime}) - h(x,y)} \right]^{2} } } dxdy $$

For a random rough surface, the height–height correlation function has the form^14,34^:

7 $$ G(r) \approx r^{{2\alpha }{}} for^{{}}\, r < < \xi $$

8 $$ G(r) \approx 2w^{{2}{}} for^{{}}\, r > > \xi $$

*R*(*r*) and *G*(*r*) are related by^14,34^:

9 $$ G(r) \approx 2w^{2} \left[ {1 - R(r)} \right] $$

where *α* is the roughness exponent.

The power spectrum or structure factor of the surface, *S(k)*, is the square of the Fourier transform of the surface height profile *h(x,y)* and can be expressed as^14,34^:

10 $$ S(k) = \frac{1}{{L^{2} }}\left| {\int_{ - L/2}^{L/2} {\int_{ - L/2}^{L/2} {\left[ {\left\{ {h(x + x^{\prime},y + y^{\prime}) - h(x,y)} \right\}e^{{ - i(k_{x} x + k_{y} y)}} } \right]} } dxdy} \right|^{2} $$

If the surface height fluctuation is periodic with a repetition length, *l*, the periodicity will be reflected in the power spectrum *S*(*k*) as a peak at the wavenumber *k* = 2*π*/*l*.

To understand the nature of the surface micro-textures in detail, extensive analysis of AFM images was carried out using the Gwyddion software ver. 2.53^34^. The second-order statistical quantities observing the mutual relationship of two points on the surface are employed in analyses^34^.

**Figure 8:**
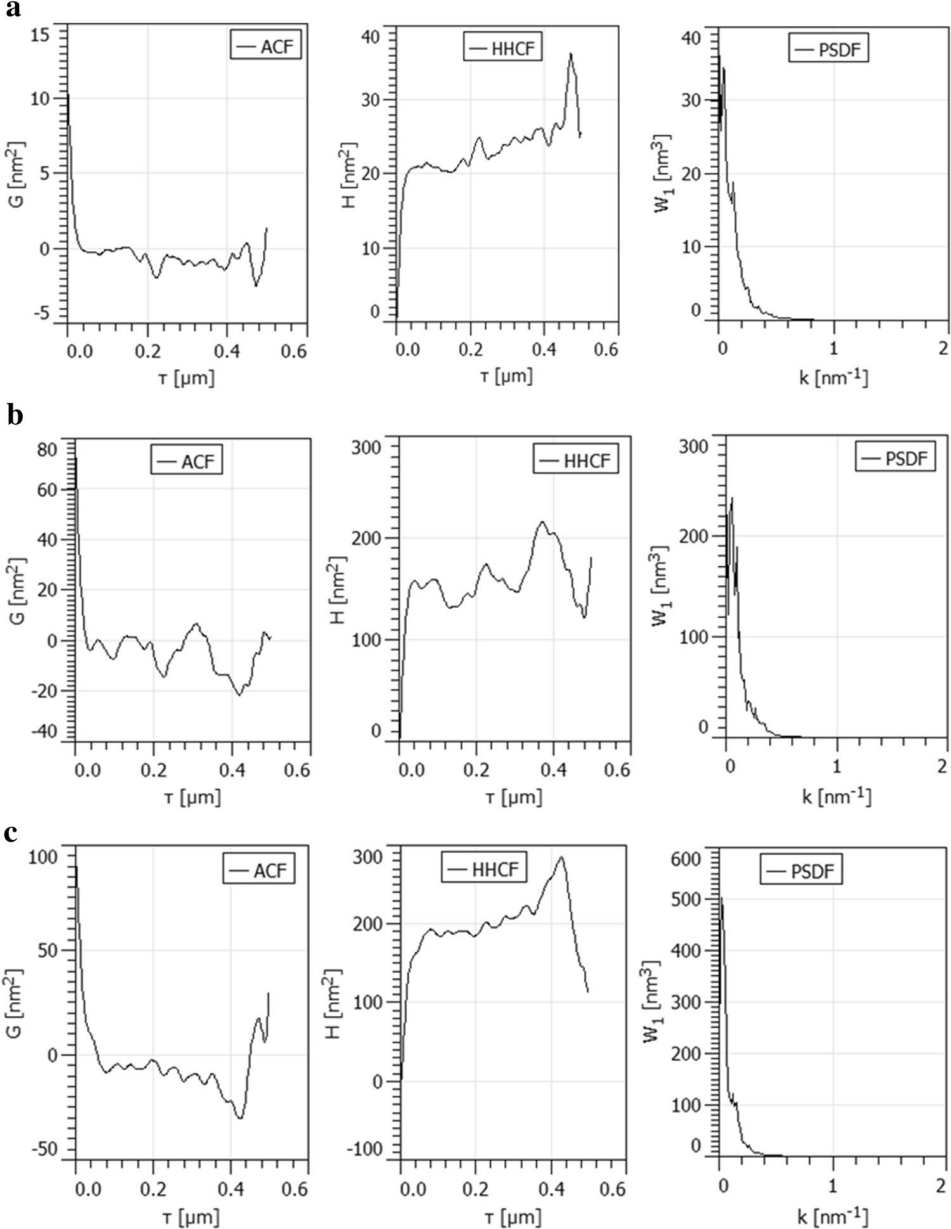
The ACF, HHCF and PSDF functions computed for samples (**a**) #1, (**b**) #2 and (**c**) #3 deposited at various times 10, 25 and 40 min (Gwyddion software, ver. 2.53, http://www.cmi.cz/).

The statistical functions (the autocorrelation function—ACF, the height–height correlation function—HHCF, the power spectral density function—PSDF) computed for samples are shown in Fig. 8.

It can be seen that the ACF and HHCF functions of all samples are characterized by the high speed of data variation. The PSDF functions for all samples have similar graphical variations. The graphical statistical functions confirm the multifractal results and these are highly correlated at this scale. In these areas, surface microtexture is heterogeneous, containing sub-regions of different micropatterns.

## Conclusions

The surface microtexture characteristics of Ag/DLC nanocomposite prepared by RF-PECVD and RF-sputtering co-deposition method was quantitatively investigated using AFM together with the 3-D surface micromorphology and multifractal analyzes. The study revealed a nanometer multifractal nature of the samples.

Statistical and multifractal analysis of the surface height function observed by the AFM experiments provides information about the development of surface morphology with the sample deposition times. Generalized fractal dimension, multifractal singularity spectra, and statistical functions show results consistent with the XRD and UV–visible spectroscopy measurements. From experimental topography data, and appropriate analyses, the effectiveness, and accuracy of our approach have been demonstrated.

AFM observation of the surfaces showed the growth of nanosized features as the deposition time increases. The multifractal analysis revealed that these objects have fractal nature which is unchanged as the deposition time rises: small fractals appear at the initial stage of plasma treatment and then become larger without altering the fractal properties. On the basis of these findings, the results presented here could have a great impact on the development of advanced materials with new potential applications in fields as diverse as sensors, tribology, and biomaterials can be found.

## Data Availability

The datasets analyzed during the current study are available from the corresponding author on reasonable request.

## Abbreviations

Ag/DLC: Silver in diamond like carbon
RF-PECVD: Radiofrequency plasma-enhanced chemical vapor deposition
AFM: Atomic force microscopy
XRD: X-ray diffraction
UV–visible: Ultraviolet–visible
LSPR: Localized surface plasmon resonance
3D: Three dimensional
NPs: Nanoparticles
FTIR: Fourier transform infrared
RMS: Root mean square
ACF: The autocorrelation function
HHCF: The height–height correlation function
PSDF: The power spectral density function
